# Importance of Choice of Drugs and Timing of Their Administration

**DOI:** 10.5812/kowsar.22287523.3549

**Published:** 2012-01-01

**Authors:** Kyung Y. Yoo, Jong Un Lee

**Affiliations:** 1 Department of of Anesthesiology, Chonnam National University of Medical School, Gwangju, South Korea; 2 Department of Physiology, Chonnam National University Medical School, Gwangju, South Korea

**Keywords:** Preeclampsia, Intubation, Nifedipine

Dear Editor,

Safavi et al. (1) examined whether hydralazine, nitroglycerin or nifedipine effectively attenuates the hemodynamic response to laryngoscopy and tracheal intubation in severe preeclamptic women undergoing cesarean delivery under general anesthesia. They found that continuous intravenous (IV) infusion of nitroglycerin was more effective in attenuating the pressor response than IV hydralazine 5-10 mg or sublingual nifedipine 10 mg without significant adverse effects on the newborn. While their study provides some important informations, we would like to point out some methodological concerns and the choice of drugs.

First, they administered drugs 3 minutes before laryngoscopy and tracheal intubation despite their different onset of action. The relatively slow onset and long duration of action of hydralazine may prevent rapid adjustment of blood pressure. Moreover, after a single sublingual administration of nifedipine 10 mg, an antihypertensive action is obvious within 5 to 8 minutes with peak effects at 20 to 30 minutes (2). Therefore, to maximize the effect, it should have been better to administer nifedipine at time point when it would exhibit its peak effect during the tracheal intubation. Indeed, Kumar et al. (3) have demonstrated that sublingual nifedipine given 20 minutes before induction of anesthesia effectively attenuates the hypertensive response to tracheal intubation in pregnancy-induced hypertension. On the other hand, nitroglycerin has rapid onset and short duration of action (4). However, mean systolic blood pressure just before the onset of tracheal intubation was similar to their baseline values in the nitroglycerin group (1), suggesting that the effect of nitroglycerin was not apparent at the time point. In contrast, in a previous study, nitroglycerin lowered mean arterial blood pressure by 20% just before tracheal intubation, and effectively attenuated the pressor response to intubation in preeclamptic women undergoing cesarean delivery (4). Nitroglycerin should have been started earlier in their study.

Second, the hypertensive response to intubation is transient, so that the duration of action of drugs should be short. With a prolonged duration of action, they may result in hypotension, as shown by a higher incidence of hypotension in those given hydralazine or nifedipine in their study (1). Moreover, opioids have been demonstrated to increase the risk of neonatal respiratory depression after cesarean delivery owing to their long duration of action (5). Remifentanil, a potent, synthetic μ-receptor agonist, has an extremely short duration of action (6). It may thus afford advantages at the induction, without subsequent neonatal depression. It has been indeed shown to attenuate the cardiovascular response to intubation with minimal neonatal respiratory depression (7).

Several pharmacologic agents, including opioids (5, 7), hydralazine, nifedipine (3), calcium channel blockers, or nitrates (4), have been tried to prevent or to control hemodynamic responses to intubation while undergoing cesarean delivery. Nevertheless, they are not always safe, convenient, or predictable. Nitroglycerin dilates cerebral vasculature (8) and may increase intracranial pressure especially in severe pre-eclamptics with cerebral edema, and crosses the placenta (4). In addition, nitroglycerin and nifedipine attenuate the pressor but not the tachycardia responses (3, 4). Owing to its high potency and ultra-short duration of action, remifentanil appears to be most reliable thus far in attenuating the cardiovascular response to intubation while undergoing cesarean delivery in both the maternal and fetal perspectives. Nevertheless, in many countries, neuraxial anaesthesia has become a preferred technique to provide anaesthesia for cesarean delivery even among women with severe pre-eclampsia (9).
